# Elevated Biomarkers of NETosis in the Serum of Pediatric Patients With Type 1 Diabetes and Their First-Degree Relatives

**DOI:** 10.3389/fimmu.2021.699386

**Published:** 2021-07-07

**Authors:** Adam Klocperk, Jana Vcelakova, Petra Vrabcova, Irena Zentsova, Lenka Petruzelkova, Zdenek Sumnik, Stepanka Pruhova, Anna Sediva, Zuzana Parackova

**Affiliations:** ^1^ Department of Immunology, 2nd Faculty of Medicine Charles University, University Hospital in Motol, Prague, Czechia; ^2^ Department of Pediatrics, 2nd Faculty of Medicine, Charles University and Motol University Hospital, Prague, Czechia

**Keywords:** type 1 diabetes, neutrophils, NETosis, PAD4, pediatric, neutrophil extracellular trap, NET, ELISA

## Abstract

Type 1 diabetes (T1D) is an autoimmune disorder with unambiguous involvement of both innate and adaptive immune mechanisms in the destruction of pancreatic beta cells. Recent evidence demonstrated that neutrophils infiltrate the pancreas prior to disease onset and therein extrude neutrophil extracellular traps (NETs), web-like structures of DNA and nuclear proteins with a strong pro-inflammatory biologic activity. Our previous work showed that T1D NETs activate dendritic cells, which consequently induce IFNγ-producing Th1 lymphocytes. The aim of this study was to assess direct *ex vivo* biomarkers of NETosis in the serum of recent onset and long-term pediatric T1D patients, their first-degree relatives and healthy controls. To this end we evaluated serum levels of myeloperoxidase (MPO), neutrophil elastase (NE), proteinase 3 (PR3), protein arginine deiminase 4 (PAD4), LL37 and cell-free DNA-histone complexes in sex- and age-matched cohorts of T1D first-degree relatives, recent-onset T1D patients, and in patients 12 months after clinical manifestation of the disease. Our data shows that disease onset is accompanied by peripheral neutrophilia and significant elevation of MPO, NE, PR3, PAD4 and cell-free DNA-histone complexes. Most biomarkers subsequently decrease but do not always normalize in long-term patients. First-degree relatives displayed an intermediate phenotype, except for remarkably high levels of LL37. Together, this report provides evidence for the presence of ongoing NETosis in pediatric patients with T1D at time of clinical manifestation of the disease, which partly subsides in subsequent years.

## Introduction

Type 1 diabetes (T1D) is an autoimmune disease resulting from the destruction of insulin-producing beta cells in the pancreas, which involves both innate and adaptive immunity. Neutrophils in particular enjoy the interest of the scientific community, with both reduced (1–3) and elevated (4, 5) neutrophil counts being reported in T1D patients. While it has been shown that neutrophils can infiltrate the pancreas and initiate the autoimmune response driving its destruction in mice (6), their precise role in the pathogenesis of T1D is still under debate.

Neutrophils are able to extrude web-like structures called neutrophil extracellular traps (NET) in a process of specific cell death called NETosis (7). Even though NETosis is an essential part of host defense against infection, it can also be involved in the pathogenesis of autoimmune diseases. We and others have shown the involvement of NETs in the pathogenesis of autoimmune diabetes (6, 8–10), where neutrophils and NET-related products act as drivers of inflammation (11), Th1 polarization and type II interferon production (10). Conversely, the inhibition of NET formation is able to attenuate the development of T1D (6).

In previous studies, increased circulating levels of neutrophil elastase (NE) and proteinase 3 (PR3), both serine proteinases produced by neutrophils and stored in their primary azurophilic granules, as well as increased levels of myeloperoxidase (MPO)-DNA complexes, were reported in patients with T1D (12), suggesting enhanced NET formation. However, another study refuted this observation and documented significantly decreased levels of NE and PR3 (9) in patients within 3 years of diagnosis, a finding possibly related to the previously shown gradual decrease of neutrophil counts after clinical onset of the disease (13).

In this study we aim to expand the spectrum of investigated NETosis related products to NE, PR3, MPO, peptidyl arginine deiminase 4 (PAD4) – an enzyme which facilitates chromatin decondensation vital for NET formation, LL37 – an antimicrobial cathelicidin, and DNA-histone complexes and assess their levels in the serum of a cohort of patients at the onset of T1D, with well-established disease and in their first-degree relatives, both autoantibody positive and negative.

## Methods

### Cohort Description

Peripheral blood neutrophil counts were measured as part of complete blood count with differential, using routine in-house methods in 333 patients with long-term T1D at an average of 5.73 ± 3.82 years since clinical manifestation of T1D, 172 patients with newly diagnosed T1D sampled within 7 days of manifestation, 51 antibody positive [at least one of the following: anti-glutamic acid decarboxylase 65 (anti-GAD65), anti-tyrosine phosphatase-like insulinoma antigen 2 (anti-IA2), anti-indole-3-acetic acid (anti-IAA), anti-zinc transporter protein 8 (anti-ZNT8)] first-degree relatives of T1D patients, 122 antibody negative first-degree relatives of T1D patients and 17 healthy children.

Serum neutrophil products were measured in a subset of 31 patients with newly diagnosed T1D [8 who presented with diabetic ketoacidosis (DKA, pH < 7.3)], 32 patients at one year since clinical manifestation of T1D. Additionally, we investigated 32 antibody negative and 32 antibody positive healthy first-degree relatives. The control group with no personal history of autoimmune disease comprised 32 age and sex-matched healthy children.

Detailed cohort description data can be inspected in **Tables 1** and **2**.

**Table 1:** Cohort description – neutrophil counts.

	Number (n)	Age (years, mean ± SD) (range)	Sex (n)	Time since diagnosis of T1D (years, mean ± SD) (range)	HbA1c (mmol/mol, mean ± SD) (range)
Healthy	17	14.29 ± 1.87	5 male, 12 female	NA	NA
		(11.81-17.37)			
Ab- relatives	122	9.11 ± 4.48	61 male, 61 female	NA	34.77 ± 2.76
		(0.3-20.7)			(27–39)
Ab+ relatives	51	9.07 ± 4.37	29 male, 22 female	NA	32.83 ± 3.57
		(2.7-25.6)			(25-40)
Recent onset	172	9.19 ± 4.44	85 male, 87 female	NA	97.63 ± 29.83
					(33-172)
		(1.1-18.27)			
Long-term T1D	333	12.61 ± 4.21	168 male, 165 female	5.73 ± 3.82	64.48 ± 15.42
		(2.28-24.58)		(0.71-17.5)	(35-143)

**Table 2:** Cohort description – serum neutrophil products.

	Number (n)	Age (years, mean ± SD)	Sex (n)	HbA1c (mmol/mol, mean ± SD) (range)
Healthy	32	9.6 ± 4.2	16 male, 16 female	NA
		(3.8-17.9)		
Ab- relatives	32	9.5 ± 4.1	16 male, 16 female	33 ± 4.1
		(2.0-17.4)		(28-43)
Ab+ relatives	32	9.9 ± 4.2	16 male, 16 female	33 ± 4.8
		(3.2-17.6)		(27-40)
Recent onset	31	9.6 ± 4.2	15 male, 16 female	106.5 ± 25.5
		(3.3-17.5)		(59-156)
Long-term T1D	32	10.1 ± 4.3	16 male, 16 female	50 ± 22.8
		(3.2-17.5)		(32-125)

Legal guardians of all study participants signed a written informed consent prior to entering the study. The study was approved by the institutional Ethics Committees of the University Hospital Motol and 2nd Faculty of Medicine, Charles University in Prague, Czech Republic and was conducted in accordance with the Declaration of Helsinki.

### Neutrophil Counts and Autoantibody Determination

Anti-GAD65, -IA2, -IAA autoantibodies were measured using radioimmunoassay (RIA) based on 125I-labelled antigens (Medipan GmbH, Berlin, Germany). All three assays were evaluated using the Islet Autoantibody Standardization Program 2015. The following assay cut-offs were determined with receiver operating characteristic (ROC) plots using all the samples: 0.4 U/ml for anti-IAA, 1.0 U/ml for anti-GAD65 and 0.9 U/mL for anti-IA2. Anti-ZnT8 were examined by ELISA (RSR Limited, Wales, UK) as described previously (14). Complete blood count with differential was analysed on the Sysmex XN-3000 platform (Sysmex Europe, Norderstedt, Germany).

### NET Components

Commercially available ELISA kits were used to quantify myeloperoxidase, neutrophil elastase, proteinase 3 (Abcam, Cambridge, USA), LL37 (Hycult Biotech, Wayne, USA), DNA-histone complexes (Sigma-Aldrich, St. Luis, USA) and PAD4 (LSBio, Seattle, USA) according to manufacturer’s instructions.

### Statistics

Statistical analyses were performed using Brown-Forsythe and Welch one-way analysis of variance (ANOVA), unpaired t-tests with Welch’s correction and linear regression using GraphPad PRISM 8.0 (San Diego, CA, USA). Values of p=0.01-0.05 (*), p=0.001-0.01 (**), p<0.001 (***) and p<0.0001 (****) were considered statistically significant.

## Results

### T1D Onset Is Accompanied by Transient Neutrophilia

**Figure 1:**
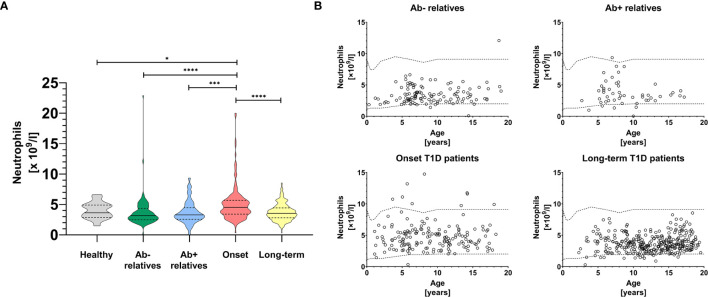
Peripheral blood neutrophils. Absolute neutrophil counts in the peripheral blood of healthy controls, antibody negative and antibody positive first-degree relatives of T1D patients, T1D patients at the clinical onset of the disease and T1D patients with long-term, well-established disease **(A)**. Temporal development of absolute neutrophil counts in the peripheral blood of healthy controls, antibody negative and antibody positive first-degree relatives of T1D patients, T1D patients at the clinical onset of the disease and T1D patients with long-term, well-established disease **(B)**. Healthy in-house reference range visualized with dashed line. Unpaired t-test with Welch’s correction p-values shown, p=0.01-0.05 (*), p=0.001-0.01 (**), p<0.001 (***), p<0.0001 (****).

In this study we observed substantial changes in absolute circulating neutrophil counts between healthy controls, first-degree relatives of T1D patients both negative and positive for T1D-specific autoantibodies, patients at clinical onset of the disease and patients with long-term disease (**Figure 1A**, Brown-Forsythe and Welch ANOVA p < 0.0001, summary data for other leukocyte populations shown in **Supplementary Table 1**).

In particular, we saw a modest but statistically insignificant decrease of neutrophils in relatives regardless of seropositivity and in patients with long-term disease, compared to healthy controls (**Figure 1A**), which was independent from age (**Figure 1B**). In contrast, patients at the onset of clinical disease had elevated absolute neutrophil counts compared to first-degree relatives (p < 0.0001 for antibody negative and p = 0.001 for antibody positive, unpaired t-test with Welch’s correction), long-term T1D patients (p < 0.0001) and healthy controls (p = 0.014).

### Increased Circulating Levels of NET-Associated Biomarkers Are a Hallmark of Recent Onset T1D Patients

Since the process of NETosis has previously been implicated in the pathogenesis of T1D and our patients displayed a discrete transient neutrophilia upon reaching clinical onset of T1D, we hypothesized that NET-associated biomarkers should be elevated in the serum of T1D patients, especially at the time of clinical manifestation of the disease.

**Figure 2:**
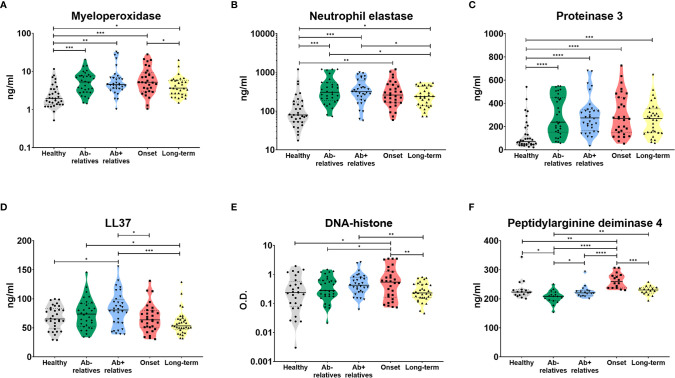
Serum products of NETosis. Products of NETosis **(A)** myeloperoxidase, **(B)** neutrophil elastase, **(C)** proteinase 3, **(D)** LL37, **(E)** DNA-histone complexes and **(F)** peptidyl arginine deiminase 4) in the serum of healthy controls, antibody negative and antibody positive first-degree relatives of T1D patients, T1D patients at the clinical onset of the disease and T1D patients with long-term, well-established disease. Unpaired t-test with Welch’s correction p-values shown, p=0.01-0.05 (*), p=0.001-0.01 (**), p<0.001 (***), p<0.0001 (****). O.D. = optical density.

Indeed, we were able to detect high levels of myeloperoxidase (MPO) (**Figure 2A**, Brown-Forsythe and Welch ANOVA p = 0.0024), neutrophils elastase (NE) (**Figure 2B**, p = 0.0005) and proteinase 3 (PR3) (**Figure 2C**, p < 0.0001), enzymes widely present in NET structures and released during the degranulation process, in the sera of T1D patients and their relatives, compared to healthy controls.

Antibody positive relatives (Ab+) also exhibited significantly elevated levels of LL37 (cathelicidin), an antimicrobial peptide commonly present in NET structures (**Figure 2D**, p = 0.0058). There was no relationship between a number of autoantibodies and NET-related products (**Supplementary Figure 1**).

Patients at the clinical onset of T1D displayed increased levels of cell-free DNA-histone complexes (**Figure 2E**, p = 0.0031) and peptidyl arginine deiminase 4 (PAD4), an enzyme which facilitates protein citrullination and has been intricately linked to NET formation (**Figure 2F**, p < 0.0001).

We did not observe significant difference in serum levels of any of the analytes and absolute circulating neutrophil counts in recent onset patients who presented with and without diabetic ketoacidosis (DKA), defined as pH < 7.3 (**Supplementary Figure 2**). MPO and DNA-histones levels significantly correlated with blood pH and MPO was slightly higher in patients with DKA but showed high variance (unpaired t-test with Welch’s correction p = 0.046).

None of the NET-associated biomarkers was significantly associated with age (**Supplementary Figure 3**) or sex (**Supplementary Figure 4**). There was also no significant correlation between their serum levels and absolute counts of circulating neutrophils (**Supplementary Figure 5**).

Residual beta-cell activity was not directly quantified through the measurement of fasting or stimulated C-peptide, however there was no correlation between the metabolic control of T1D measured as glycated hemoglobin fraction HbA1c (**Supplementary Figure 6**) and NET-associated biomarkers in either recent onset or long-term patients.

## Discussion

In this brief report we demonstrate the elevation of neutrophils and indirect serum biomarkers of neutrophil NETosis – the enzymes myeloperoxidase (MPO), proteinase 3 (PR3), neutrophil elastase (NE) and protein arginine deiminase 4 (PAD4), the active form of the antimicrobial peptide cathelicidin, LL37, and cell-free DNA-histone complexes – in the blood of pediatric patients with recent onset type 1 diabetes.

Our study builds on and expands the previous works by Wang et al., who have shown similar results, but whose analysis was limited to PR3 and NE (12). We show for the first-time elevated serum levels of cell-free DNA and MPO. At the same time, the elevated concentration of PAD4 provides mechanistic insight into the process of NETosis in diabetic patients, as PAD4 citrullinates arginine residues on histones, reducing their positive charge and allowing chromatin decondensation vital for NET formation (15). LL37, which apart from its antimicrobial activity can suppress neutrophil apoptosis (16), was highest in antibody positive relatives, but quite normal in recent onset patients, suggesting concurrent activity of several pathways involving neutrophils.

The data concerning neutrophils and NETosis-biomarkers in T1D are not homogeneous. A study by Qin et al. (9) reported reduced NE and PR3 in T1D patients, which was associated with decreased neutrophils. While we too observed some correlation between MPO, NE, LL37 and neutrophil counts in T1D patients, this was not apparent in first-degree relatives and was driven by a single data point in each cohort. A possible explanation for these diverging results are the different inclusion criteria, as Qin’s “recent-onset” cohort included patients up to 3 years after disease manifestation and featured mainly adults, whereas our recent onset cohort was sampled within 7 days of the clinical manifestation of the disease and comprised chiefly children under 10 years of age. A closer comparison may be drawn to the study by Valle et al., which reported decreased neutrophil counts in newly diagnosed pediatric T1D patients (1). The potential effect of recent metabolic stress on circulating neutrophils and NETosis cannot be discounted and presents a unique and currently unresolved challenge in determining which of these two is the initial driving factor, which will require further study. The slight neutropenia we reported in first-degree relatives is in agreement with previous observations by Vecchio et al. (2), suggesting that despite the absence of overt endocrinopathy, abnormalities in neutrophil biology are already present in at risk subjects. Further work on neutrophil phenotype in recent onset patients and at-risk relatives is warranted and could elucidate their activation status, maturity and more.

While we have already previously shown that T1D NETs activate dendritic cells and drive T cell polarization towards the IFN-γ Th1 response in a series of *in vitro* studies performed on material from patients with well-established disease (10), here for the first time we show NETosis biomarkers directly *ex vivo* and expand the studied cohorts to include recent onset patients and their relatives. As the current literature lacks any data analyzing the enzymatic activity of MPO, PAD4 and other enzymes, or the serum concentration of NET-derived citrullinated proteins in T1D patients, this report provides first indirect evidence of their role in T1D pathogenesis.

The double-edged role of NETosis in driving not only antimicrobial host defense, but also pathological inflammation, remains highly topical and has recently been shown in COVID-19, where elevated neutrophil counts predicted worse clinical outcome and serum MPO and cell-free DNA were elevated in patients requiring mechanical ventilation (17). The mass egress of proteolytic enzymes from neutrophils into circulation can trigger a proteolytic storm, drive activation of pro-enzymes and result in proinflammatory cytokine release and host damage (18).

We thus hypothesize that a yet unidentified trigger, perhaps a subclinical viral infection (19), contributes to the ongoing neutrophil activation and low-grade NETosis that we show already in antibody-positive relatives. The biological activity of NETs may then result in further accentuation of the inflammation, egress of neutrophils from the bone marrow – reflected in the transient neutrophilia we show – and neutrophil infiltration into the pancreas as documented by Diana et al. Ultimately, this vicious cycle leads to the destruction of insulin-producing beta-cells and clinical manifestation of diabetes mellitus.

## Data Availability Statement

The raw data supporting the conclusions of this article will be made available by the authors upon request.

## Ethics Statement

The studies involving human participants were reviewed and approved by the institutional Ethics Committees of the University Hospital Motol and 2nd Faculty of Medicine, Charles University in Prague. Written informed consent to participate in this study was provided by the participants’ legal guardian/next of kin.

## Author Contributions

AK gathered and analyzed longitudinal data mapping peripheral neutrophil counts and co-wrote the article. PV and IZ performed experiments. JV, LP, ZS, and SP provided patient information and primary biomaterial and reviewed the manuscript. AS co-conceived the study, provided support and reviewed the manuscript. ZP conceived the article, designed experiments evaluating the serum concentration of NETosis products, analyzed the data and co-wrote the article. All authors contributed to the article and approved the submitted version.

## Funding

The work was supported by AZV NU 20-05-00320 and NU20-05-00282 issued by the Czech Health Research Council and Ministry of Health, Czech Republic and institutional support of research organization #00064203 from University Hospital in Motol, Czech Republic.

## Conflict of Interest

The authors declare that the research was conducted in the absence of any commercial or financial relationships that could be construed as a potential conflict of interest.
