# Supplementary material for: Elevated Biomarkers of NETosis in the Serum of Pediatric Patients With Type 1 Diabetes and Their First-Degree Relatives
Source: Front Immunol. 2021 Jul 7;12:699386. doi: 10.3389/fimmu.2021.699386 (PMC8293100; doi:10.3389/fimmu.2021.699386)
Supplement: Supplementary file 1 [file DataSheet_1.docx]

Supplementary Material

# Supplementary Figure 1 NET-associated biomarkers × number of autoantibodies

# Supplementary Figure 2 NET products in sera of patients with and without diabetic ketoacidosis

Serum concentration of neutrophil elastase, proteinase 3, LL37, DNA-histone complexes, peptidyl arginine deiminase 4 and absolute neutrophil counts were not significantly different between recent onset patients with and without diabetic ketoacidosis (DKA). MPO was somewhat higher in patients with DKA (unpaired t-test with Welch’s correction p = 0.046). Correlation between NET-associated biomarkers and blood pH in recent onset T1D patients. O.D optical density


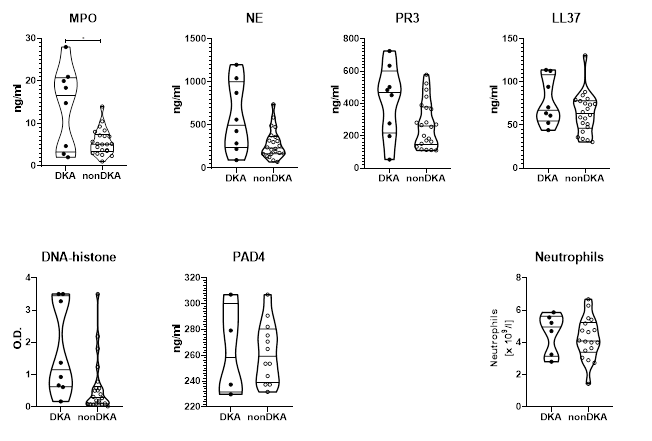


# Supplementary Figure 3 Correlation of serum NET products with age

Serum concentration of myeloperoxidase, neutrophil elastase, proteinase 3, LL37, DNA-histone complexes and peptidyl arginine deiminase 4 does not correlate significantly with age in a population of healthy children and adolescents. Linear regression trendlines and 95% confidence bands shown, with p and R values.

# Supplementary Figure 4 NET-associated biomarkers products in males and females

There is no significant association between serum concentration of NET-associated biomarkers and sex.

# Supplementary Figure 5 NET-associated biomarkers × absolute neutrophil count

Correlation between NET-associated biomarkers and absolute neutrophil counts in recent onset and long-term T1D patients, as well as their first-degree relatives.


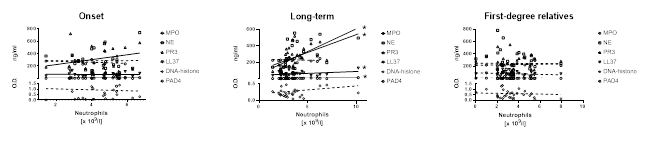


# Supplementary Figure 6 NET-associated biomarkers × HbA1c

No correlation was found between serum NET-associated biomarkers and HbA1c.


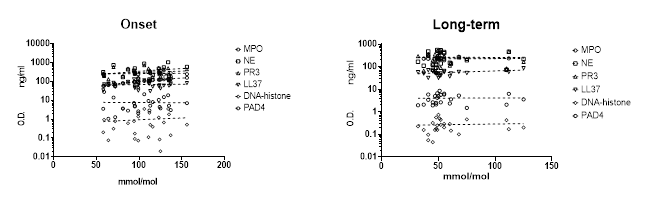


# Supplementary Table 1 Leukocyte subpopulations

|  | Lymphocytes  (×10^9^/l, mean±SD) (range) | Monocytes  (×10^9^/l, mean±SD) (range) | Neutrophils  (×10^9^/l, mean±SD) (range) | Eosinophils  (×10^9^/l, mean±SD) (range) | Basophils  (×10^9^/l, mean±SD) (range) |
| --- | --- | --- | --- | --- | --- |
| Healthy | 2.6±0.4  (1.9-3.3) | 0.6±0.2  (0.4-0.9) | 4.0±1.4  (1.5-6.6) | 0.32±0.38  (0.04-1.6) | 0.04±0.01  (0.02-0.07) |
| Ab- relatives | 2.7±1.0  (0.8-10.2) | 0.7±0.2  (0.2-2.4) | 3.6±2.2  (0.3-20.0) | 0.29±0.29  (0,0-1,5) | 0.014±0.01  (0.0-0.04) |
| Ab+ relatives | 2.4±1.0  (1.3-5.2) | 0.7±0.2  (0.3-1.3) | 3.7±2.2  (0.0-9.4) | 0.08±0.19  (0.1-1.4) | 0.037±0.02  (0.0-0.11) |
| Recent onset | 3.0±1.3  (0.8-10.2) | 0.7±0.3  (0.2-2.4) | 5.0±2.8  (0,3-20.0) | 0.17±0.23  (0.0-0.7) | 0.015±0.02  (0.0-0.16) |
| Long-term T1D | 2.4±0.8  (0.4-5.4) | 0.6±0.2  (0.3-1.6) | 3.7±1.4  (0.3-8.6) | 0.25±0.25  (0.1-1.2) | 0.037±0.02  (0.0-0.16) |
